# Probiotic Properties of Lactic Acid Bacteria Isolated From Neera: A Naturally Fermenting Coconut Palm Nectar

**DOI:** 10.3389/fmicb.2019.01382

**Published:** 2019-06-28

**Authors:** Rakesh Somashekaraiah, B. Shruthi, B. V. Deepthi, M. Y. Sreenivasa

**Affiliations:** ^1^Department of Studies in Microbiology, University of Mysore, Mysuru, India; ^2^Department of Biotechnology, Sahyadri Science College, Kuvempu University, Shimoga, India

**Keywords:** lactic acid bacteria, Neera, probiotics, bio-preservatives, gastrointestinal tract

## Abstract

Probiotic bacteria were isolated from different traditional fermented foods as there are several such foods that are not well explored for their probiotic activities. Hence, the present study was conducted to find the potential of lactic acid bacteria (LAB) as probiotics that were isolated from the sap extract of the coconut palm inflorescence – Neera, which is a naturally fermented drink consumed in various regions of India. A total of 75 isolates were selected from the Neera samples collected aseptically in the early morning (before sunrise). These isolates were initially screened for cultural, microscopic, and biochemical characteristics. The initial screening yielded 40 Gram-positive, catalase-negative isolates that were further subjected to acid – bile tolerance with resistance to phenol. Among 40 isolates, 16 survived screening using analysis of cell surface hydrophobicity, auto aggregation with adhesion to epithelial cells, and gastric–pancreatic digestion for gastrointestinal colonization. The isolates were also assessed for antimicrobial, antibiotic sensitivity, and anti-oxidative potential. The safety of these isolates was evaluated by their hemolytic and deoxyribonuclease (DNase) activities. Based on these results, seven isolates with the best probiotic attributes were selected and presented in this study. These LAB isolates, with 51.91–70.34% survival at low pH, proved their resistance to gastric conditions. The cell surface hydrophobicity of 50.32–77.8% and auto aggregation of 51.02–78.95% represented the adhesion properties of these isolates. All the seven isolates exhibited good antibacterial and antifungal activity, showing hydroxyl-scavenging activity of 32.86–77.87%. The results proved that LAB isolated from Neera exhibited promising probiotic properties and seem favorable for use in functional fermented foods as preservatives.

## Introduction

The lactic acid bacteria (LAB) are isolated from various food matrices and those isolates with better performances and high competitiveness are used as probiotics (Bromley and Uchman, 2003). The probiotics are live organisms that confer health benefits on the host when consumed in adequate amounts, by bringing the microbial balance in the system. The use of probiotics in animal infections, especially in the gastrointestinal and vaginal tract, has been extensively studied (Pineiro and Stanton, 2007). They are proficient in inhibiting the growth of pathogenic organisms through different mechanisms such as adherence to epithelial cells, modulation of the immune system, and secretion of antimicrobial compounds. This proves their ability in the bio-preservation of food and can be used as starter culture in the fermentation process under controlled conditions. Therefore, the isolation and characterization of LAB from different traditional fermented foods and products have gained research interest in recent years (Alonso et al., 2018).

Neera is a sap extract, a naturally fermented drink collected by tapping the spadix of coconut palm. This coconut palm inflorescence sap extract is consumed before sunrise in India, Sri Lanka, Malaysia, Indonesia, Myanmar, and Thailand as a sweet juice. Neera with a pH of 6.5–7.0 is rich in carbohydrates and highly nutritional and helps in digestion (Borse et al., 2006). It is naturally fermented by different microorganisms among which LAB are one of the protagonists. Neera also possesses medicinal properties because of which it is used as syrup in Indian system of Ayurveda preparations. These factors prove that LAB isolates from Neera have potential probiotic properties (DebMandal and Mandal, 2011).

Neera obtained from the spadix of coconut palm undergoes natural fermentation due to the innate presence of microorganisms like yeasts and bacteria, resulting in the production of ethyl alcohol. The amount of alcohol production or the fermentation time depends on the storage time after Neera collection, and the process of fermentation gets enhanced under sunlight (Xia, 2011). The fresh Neera collected under hygienic conditions before sunrise and transferred at low temperatures is used for analysis. The composition of Neera is influenced by the place, time, and duration of tapping. It mainly contains total sugars, reducing sugar, ethanol, volatile acids, amino acids, vitamins, and phenolic compounds (Borse et al., 2006; Chinnamma et al., 2019).

A potent probiotic isolate must possess certain characteristics like survival and colonizing ability under different environmental conditions (Palachum et al., 2018). The isolates should be able to withstand low pH of gastric juice with resistance to bile salts and also adhere to epithelial cells. They should also offer certain health benefits like antimicrobial activity, anticancer activity, toxin-reducing effects, and boosting immune response. Hence, bacteria adhering to suitable surfaces and survival in the gastrointestinal tract should be confirmed by *in vitro* evaluation prior to using them as probiotics (Chiang and Pan, 2012; Berardi et al., 2013). The health benefits of LAB as probiotics such as lowering the risk of diseases, regulation of allergic response, and improving inhabitants of gastrointestinal tract with better immune response have been reported. The contribution of LAB in inhibiting the growth of pathogenic organisms, reducing their toxin secretions, and increasing the nutritional value and other functionality has already been studied (Bartkiene et al., 2018).

The LAB strains Enterococcus spp., Lactococcus spp., and Lactobacillus spp. that have antimicrobial properties are used in bio-control strategies such as reducing mycotoxins and enhancing bioavailability (Campagnollo et al., 2016; Deepthi et al., 2017). Recent studies have revealed that probiotic LAB strains can be used to remove mycotoxins such as aflatoxins, trichothecenes, and fumonisins from different food products during pre-harvest, production, and storage (Deepthi et al., 2016; Poornachandra Rao et al., 2017). These LAB strains, isolated from traditional fermented foods, can be used in the formulation of fermented foods with functional characteristics to manage the growth of adverse pathogenic microorganisms; this would help in the prevention and/or treatment of diseases in consumers (Batista et al., 2017).

The main objective of the current work was to report on the isolation of potential probiotic LAB from hitherto unexplored, naturally fermenting product – Neera. The isolated LAB strains were characterized by *in vitro* tests for their probiotic properties (antimicrobial strains surviving gastrointestinal conditions) in order for them to be used in the preservation and fermentation of food.

## Materials and Methods

### Isolation of LAB

The isolation of LAB strains from the Neera (naturally fermented nectar of coconut palm) samples was based on the method described by Poornachandra Rao et al. (2015). Briefly, fresh Neera samples were collected under hygienic conditions before sunrise from different regions of Mysuru (Karnataka, India) and transferred at low temperatures for analysis; about 1 ml of each sample was enriched in a de Man, Rogosa, and Sharpe (MRS) broth and incubated under anaerobic conditions at 37°C for 24 h. The enriched broth samples were diluted using phosphate buffer saline (PBS), plated on an MRS agar medium, and then incubated under anaerobic conditions at 37°C for 24 h. The morphologically discrete colonies were further sub-cultured onto MRS agar plates. The viable cultures were stored in MRS slants at 4°C. The stock cultures were maintained at −20°C in glycerol stock for further analysis.

### Primary Characterization of LAB Strains

Preliminary identification of the 40 LAB isolates was based on their phenotypic and biochemical characteristics that included Gram’s reaction, catalase test, nitrate reduction assay, citrate utilization assay, bile salt hydrolase activity, osmotic stress (sodium chloride: 3, 5, and 7%) resistance, and sugar fermentation ability (of isolates assimilating different sugar supplements) (Boone et al., 2001; Ni et al., 2015). The growth of the isolates at different temperatures and pH levels was also tested. The cell viability of the isolates was assessed by the plate count method and the results were presented as log colony-forming units (CFU) per milliliter (Ni et al., 2015).

### Evaluation of Probiotic Properties

#### Tolerance to Acids and Bile Salts

The tolerance of the LAB isolates to both acidic pH value and bile salts was studied using the methodology described by Guo et al. (2009) and Ramos et al. (2013). Overnight cultures of the strains were inoculated in MRS broth, initially adjusted to pH value 2.0 using 1 N hydrochloric acid (HCl) and MRS medium supplemented with 0.3% oxgall. The MRS broth adjusted to an initial pH of 6.5 was considered as the control for acidic pH value and the one without oxgall was considered as the control for bile salt condition. The samples were incubated anaerobically at 37°C for time intervals 0, 2, and 4 h and retrieved for enumeration at respective end points. The biomass (CFU/ml) of each culture obtained in the assays, made in triplicate, was enumerated on MRS agar incubated anaerobically at 37°C for 24 h. The survival rate (%) was calculated using the following formula: Survival rate (%) = Biomass at time (*t*)/Biomass at initial time (0) × 100.

### Assessment of Antibacterial Activity

The antibacterial activity of the LAB isolates was determined, using the microplate assay, against *Escherichia coli* (ATCC 25922), *Pseudomonas aeruginosa* (ATCC 15422), *Salmonella typhi* (ATCC 27870), and *Staphylococcus aureus* (ATCC 6538). The overnight cultures of LAB isolates were centrifuged (at 8,000 rpm for 10 min at 4°C), and the supernatant was filtered through a syringe filter (of 0.2-mm pore size). The prepared cell-free supernatant (CFS) was divided into two parts, one with their initial acidic pH. The rest of the CFS samples (nCFS) was neutralized to pH 6.5 using 5 M NaOH in order to eliminate the presumed effect of organic acids and both the samples stored at −20°C for further analysis.

A sterile 96-well plate was filled with 50 μl of CFS/nCFS and 50 μl of bacterial suspension to obtain ∼10^8^ CFU per well, which was made up to 200 μl using a Luria–Bertani (LB) broth. The LB broth with bacterial suspension was considered as the positive control and the LB broth alone was considered as the negative control. The plates were incubated at 37°C for 24 h, and the optical density (OD) at 600 nm was measured. The total percent inhibition of bacterial growth was calculated using the following formula: [(OD of test sample - OD of control)/OD of control] × 100 (Georgieva et al., 2015).

### Assessment of Antifungal Activity

The antifungal activity of the LAB isolates was determined, by the agar overlay method, against *Fusarium graminearum* (MTCC 1893), *Aspergillus flavus* (MTCC 2799), and *Fusarium oxysporum* (MTCC 1755). The MRS agar plates were used for the assay, on which LAB isolates were streaked at two different equidistant spots and incubated anaerobically at 37°C for 24 h. Then, 20 μl of spore suspension (∼10^6^ spores/ml) of each fungal pathogen was evenly mixed with 0.7% soft potato dextrose agar (PDA) and overlaid on the LAB-spotted MRS agar plates. The plates, after incubating aerobically at 28 ± 2°C for 4 days, were examined for clear inhibitory zones around the spot area of the LAB colonies and are tabulated (Poornachandra Rao et al., 2017).

### Molecular Identification of LAB Strains

The molecular identification of efficient LAB isolates was determined by 16S rDNA sequencing. On the basis of their potential probiotic attributes, the isolates were subjected to DNA (deoxyribonucleic acid) isolation and PCR (polymerase chain reaction) amplification using the universal primers 27F-5′AGAGTTTGATCCTGGCTCAG3′ and 1492R-5′GGTTACCT TGTTACGACTT3′. The reaction was carried out in 25 μl of the reaction mixture containing dNTP (0.2 mM), 0.5 μl of DNA template (50–100 ng), forward and reverse primers (10 pmol), 1 × PCR buffer with MgCl_2_ (magnesium chloride), and Taq polymerase (0.5 U). The optimum conditions for PCR involved an initial denaturation step for 5 min at 95°C followed by 35 cycles of denaturation for 1 min at 95°C, annealing for 1 min at 55°C, extension for 5 min at 72°C, and final extension for 7 min at 72°C. The PCR products were confirmed on agarose gel (1%) electrophoresis. Five microliters of the PCR product was loaded with 3 μl of loading dye. The PCR products were sequenced; the sequences obtained were compared using BLAST (basic local alignment search tool) and submitted to the GenBank sequence database for accession numbers (Boubezari et al., 2018).

### Antibiotic Sensitivity

The antibiotic susceptibility of the LAB isolates was assessed on MRS agar plates using the antibiotic disc diffusion method using the range of antibiotics suggested as per EFSA guidelines. The MRS agar medium was poured and allowed to solidify at room temperature. The overnight LAB cultures (100 μl) were spread on MRS agar plates and allowed to dry. The antibiotic discs were placed on the inoculated plates and incubated at 37°C for 48 h. The antibiotic susceptibility pattern of the isolates was assessed using ampicillin (10 μg/disc), vancomycin (30 μg/disc), gentamicin (10 μg/disc), kanamycin (30 μg/disc), streptomycin (10 μg/disc), chloramphenicol (30 μg/disc), erythromycin (15 μg/disc), clindamycin (2 μg/disc), and tetracycline (30 μg/disc) (Singh et al., 2012). The diameter of the zone of inhibition was measured using the antibiotic zone scale (CLSI scale). The results obtained are presented in terms of susceptibility, moderate susceptibility, or resistance. These results were compared with the interpretative zone diameters as described in Performance Standards for Antimicrobial Disc Susceptibility Tests (CLSI, 2016).

### Resistance to Phenol

The resistance of the LAB isolates to phenol was determined as described by Jena et al. (2013) with slight modifications. The MRS broth supplemented with 0.4 and 0.6% v/v phenol was inoculated with overnight-grown LAB cultures. After incubation at 37°C for 24 h, the cultures were serially diluted and spread on MRS agar plates. The cell viability (log CFU/ml) was calculated by the plate count method.

### Auto Aggregation

The ability of the LAB isolates to auto aggregate was tested as per Zommiti et al. (2017). The overnight culture was harvested by centrifugation (at 8,000 rpm at 4°C for 10 min) and washed with PBS twice and resuspended in PBS buffer. The sample was allowed to stand awhile, incubating anaerobically at 37°C, and the upper suspension was checked for absorbance at 600 nm at time intervals of 0, 1, 2, 3, 4, and 5 h. The auto aggregation was measured (in percentage) using the formula auto aggregation % = [1 − (*A*_time_/*A*_0_) × 100], where, *A*_time_ represents the absorbance at a particular time and *A*_0_ represents the absorbance at time 0.

### Cell Surface Hydrophobicity

The *in vitro* bacterial cell surface hydrophobicity of LAB isolates was evaluated by measuring the microbial cell adhesion to hydrocarbons according to the method described by Rokana et al. (2018). The overnight cultures in MRS broth were harvested by centrifugation (at 8,000 rpm at 4°C for 10 min), washed twice with PBS, and resuspended in PBS buffer followed by absorbance (*A*_0_) measurement at 600 nm. A cell suspension of about 3 ml was blended with 1 ml of hydrocarbon (xylene) and incubated at 37°C without shaking for 1 h for separation of the aqueous and organic phases. The aqueous phase (1 ml) was removed carefully and the absorbance (*A*_1_) was measured at 600 nm. The percent hydrophobicity was measured by a decrease in absorbance and calculated using the following formula: % cell surface hydrophobicity = (1 − *A*_1_/*A*_0_) × 100.

### *In vitro* Adhesion to Chicken Crop Epithelial Cells

The *in vitro* potential of the LAB isolates to adhere to epithelial cells was determined using chicken crop epithelial cells that were processed as described by Jakava-Viljanen and Palva (2007). The chicken crop was maintained in PBS at 4°C for 30 min and washed thrice with potassium phosphate buffer (pH 7.4) to remove the surface mucus. The chicken crop tissues were gently scraped using a sterile cover slip to obtain the epithelial cells and then suspended in PBS. These epithelial cells were washed twice gently using PBS by pipetting and examined under a microscope to ensure the elimination of adhering commensal bacteria. The cells were then diluted to approximately 5 × 10^6^ cells/ml. About 100 μl of LAB isolates (10^6^ CFU/ml) in 400 μl of epithelial cells was mixed well followed by incubation at 37°C for 30 min in a water bath. After incubation, the mixture was centrifuged at 3000 rpm for 3 min and then the pellet was washed twice with sterile PBS to remove non-adherent bacteria. It was then resuspended in 100 μl of PBS, stained with crystal violet, and observed under a microscope. The bacterial adhesion was examined in 10 microscopic fields and scored positive if a minimum of 10 bacteria were found adhering to each epithelial cell (Feng et al., 2018).

### Survival in Simulated Gastric and Pancreatic Digestion

The survival of LAB isolates under gastric and pancreatic conditions was determined *in vitro* by simulation. The simulated gastric juice (SGJ) and simulated pancreatic juice (SPJ) were prepared as per Lo Curto et al. (2011) with slight modifications. Then, the SGJ was mixed with pepsin (0.0133 g/L) and lysozyme (0.01 g/L) prior to use. The overnight culture was harvested by centrifugation (at 8,000 rpm at 4°C for 10 min), washed with PBS twice, and suspended with SGJ to a final absorption OD of 1.2 at 600 nm. The samples were then incubated at 37°C for 3 h in an orbital shaker at ∼200 rpm to simulate peristaltic movement. The 0- and 3-h samples were collected, serially diluted, plated on to LB agar plates for microbial counting, and incubated at 37°C for 24 h. The survival percentage in gastric juice was calculated using the following formula: g % = Tg3/Tg0 × 100, where Tg0 is the bacterial count at 0 h and Tg3 is the bacterial count at 3 h.

The LAB, incubated in SGJ for 3 h, were harvested by centrifugation (at 8,000 rpm at 4°C for 10 min), washed with PBS, and resuspended in the same volume of SPJ. The sample was plated immediately for bacterial count at time 0 h (Tp0) and then incubated at 37°C for 24 h with continuous shaking at 200 rpm. After 24 h of incubation, the sample was serially diluted and plated on LB agar plates to determine the pancreatic digestion-survived bacterial count (Tp^*^). The survival percentage in pancreatic juice was determined using the following formula: p % = Tp^*^/Tp0 × 100. The overall digestion-survival ability of the LAB isolates was estimated by S % = Tp^*^/Tg0 × 100.

### Safety Evaluation of LAB Strains

#### Hemolytic Activity

The hemolytic activity of the LAB isolates was determined using the procedure described by Yadav et al. (2016). All the isolates tested were streaked onto blood agar plates containing 5% (w/v) sheep blood and incubated at 37°C for 48 h. After incubation, the plates were examined for β-hemolysis, α-hemolysis, and non-hemolytic activities.

#### DNase Activity

The LAB isolates were streaked onto a deoxyribonuclease (DNase) agar medium to test for production of the DNase enzyme. The plates were then incubated at 37°C for 48 h and observed for the zone of DNase activity. A clear pinkish zone around the colonies was considered as positive DNase activity (Shuhadha et al., 2017).

#### Antioxidant Assay

The antioxidant assay was conducted as described by Wang et al. (2009). About 1.0 mL of 1,10-phenanthroline (0.75 mmol^–1^), 2.0 ml of sodium phosphate buffer (pH 7.4), and 1.0 ml of ferrous sulfate (FeSO_4_) (0.75 mmol^–1^) were mixed thoroughly. To the mixture, 1.0 ml of 10^9^ CFU/ml culture was added; 1.0 ml of hydrogen peroxide (H_2_O_2_) (0.01% v/v) was added to initiate the reaction and the mixture was incubated at 37°C for 90 min. After incubation, centrifugation (at 9,000 rpm for 10 min at 4°C) was carried out and the absorbance of the supernatant was measured at 536 nm. The percentage of hydroxyl radical scavenging was calculated using the following formula: hydroxyl radical scavenging activity (%) = (*A*_s_ − *A*_c_)/(*A*_B_ − *A*_c_) × 100, where *A*_s_ is the absorbance of the test sample; *A*_c_ is the absorbance of the control including 1,10-phenanthroline, FeSO_4_, and H_2_O_2_; and *A*_B_ is absorbance of the blank including 1,10-phenanthroline and FeSO_4_.

## Results

### Preliminary Characterization of LAB Isolates

A total of 75 bacterial cultures were isolated and initially subjected to physiological and biochemical tests. Out of this, 40 isolates were Gram-positive, non-spore-forming, and catalase-negative and were considered for testing as presumptive LAB isolates. The biochemical characterization revealed that the bacterial isolates were able to ferment all the tested sugars. About 16 LAB isolates showed optimum growth and also sustained osmotic stress at different NaCl concentrations. The results are presented in Table 1. The growth of these LAB isolates at different temperatures and salt conditions was also tested (Table 1). Out of 16 isolates, the data for seven isolates (i.e., MYSN 10, MYSN 106, MYSN 43, MYSN 109, MYSN 98, MYSN 18, and MYSN 28) proving their *in vitro* potential as probiotics with good antimicrobial activities are further presented.

**TABLE 1:** Phenotypic characteristics and fermentation ability of representative LAB strains isolated from Neera samples.

**Tests**	**Groups**				
	**Group A**	**Group B**	**Group C**	**Group D**	**Group E**
No. of isolates	24	5	6	4	1
Grams character	+	+	+	+	+
Shape	Cocci	Cocci	Rod	Short rods	Cocci
Catalase test	−	−	−	−	−
Citrate utilization test	−	−	−	−	−
Bile salt (0.3%) hydrolysis	+	+	+	+	+
Growth in NaCl					
3.0%	+	+	+	+	+
5.0%	+	+	+	+	+
7.0%	−	+	+	+	+
Growth at different temperatures					
4°C	+	−	+	+	+
10°C	+	−	+	+	+
37°C	+	+	+	+	+
45°C	+	+	+	+	+
Fermentation type	Homo	Homo	Homo	Homo	Hetero
Carbohydrate fermentation					
Glucose	+	+	+	+	+
Lactose	+	+	+	+	+
Sucrose	+	+	+	+	+
Xylose	+	+	+	+	+
Dextrose	+	+	+	+	+
Mannose	+	+	+	+	+
D-arabinose	−	−	−	−	−
α-Cellulose	−	−	−	−	−
Sorbitol	+	+	+	+	+
D-raffinose	−	+	+	+	+

*+, positive; −, negative; homo, homofermentative; hetero, heterofermentative.*

### *In vitro* Testing for Probiotic Potential of LAB Isolates

#### Acid and Bile Tolerances

**FIGURE 1:**
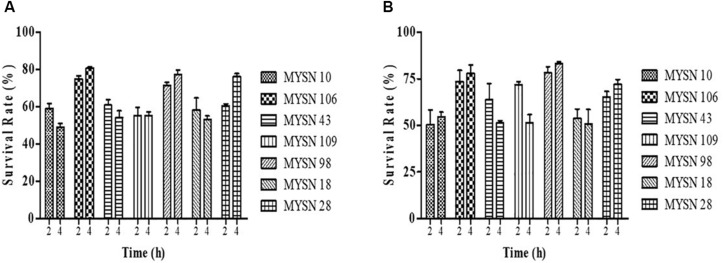
Survival rate of LAB strains isolated from Neera samples with an acidic pH value and under bile salt conditions at 37°C. **(A)** Survival of the isolates under acidic pH conditions for 2 and 4 h at 37°C in MRS agar plates. **(B)** Survival of the isolates under 0.3% bile salt conditions for 2 and 4 h at 37°C in MRS agar plates. Data shown are mean ± SD of triplicate values of independent experiments.

The acid tolerance helps in studying the survival of strains under low pH gastric juice conditions. This ability of the strains to survive the acidic pH value after 2 and 4 h of incubation at 37°C is presented in Figure 1A. The bile salt tolerance helps in the *in vitro* evaluation of metabolic activity and colonization of isolates in the small intestine. This survival of the strains in bile salts after 2 and 4 h of incubation at 37°C is presented in Figure 1B. The LAB strains were found to have a survival rate (%) above 50% at low pH and 0.3% bile salt concentration after 4-h exposure. Among all the isolates tested, the isolate MYSN 106 with a survival rate of 80.84% at low pH and MYSN 98 with 83.17% survival rate in bile proved to be the best.

#### Antagonistic Activity of LAB Isolates

**FIGURE 2:**
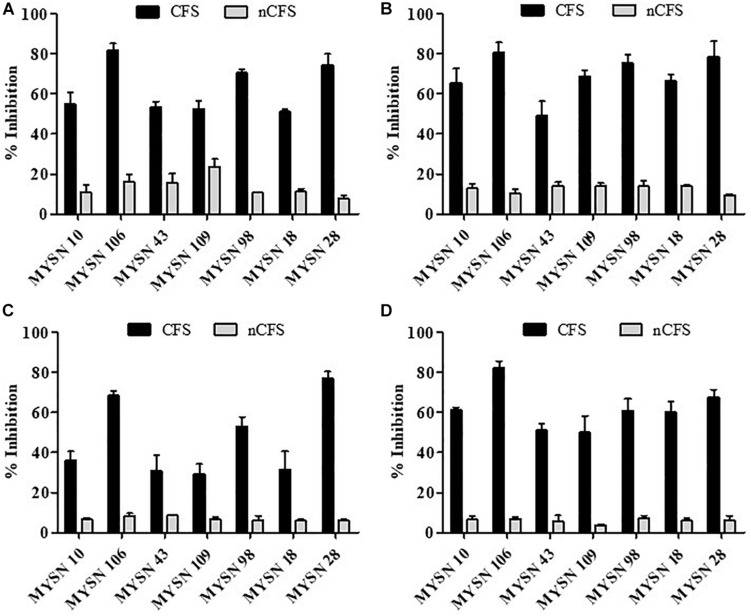
Antibacterial activity of cell-free supernatants of LAB strains isolated from Neera samples against indicator strains presented as percent inhibition. **(A)** Inhibition of *Escherichia coli.* **(B)** Inhibition of *Pseudomonas aeruginosa*. **(C)** Inhibition of *Salmonella typhi*. **(D)** Inhibition of *Staphylococcus aureus*. Data shown are mean ± SD of triplicate values of independent experiments. Note: CFS, cell-free supernatant; nCFS, cell-free supernatant neutralized to pH 6.5.

The antagonistic activity of the LAB isolates against enteric bacterial pathogens was tested. The isolates proved to have significant antibacterial activity against all the enteric pathogens. The seven selected isolates (i.e., MYSN 10, MYSN 106, MYSN 43, MYSN 109, MYSN 98, MYSN 18, and MYSN 28) showed an inhibitory effect toward the tested pathogens (Figure 2). The CFS of the isolate MYSN 106 showed 81.84, 80.81, and 82.14% inhibition of *E. coli*, *P. aeruginosa*, and *S. aureus*, respectively, and MYSN 28 inhibited about 77.10% of *S. typhi* in comparison to the other isolates tested. The activity of the CFS after neutralization to pH 6.5 (nCFS) showed minimal activity against all the pathogens tested, proving the role of organic acids for their antimicrobial activity.

The antifungal activity of the isolates was tested against *F. graminearum*, *A. flavus*, and *F. oxysporum.* Among the LAB isolates tested for 7 days, MYSN 106 showed the highest zone of inhibition: 21.25 ± 0.45 and 10.45 ± 0.70 mm against *F. graminearum* and *A. flavus*, respectively. The highest inhibition against *F. oxysporum* was shown by MYSN 28 with a 24.95 ± 0.35 mm inhibition zone. The MYSN 109 and MYSN 18 isolates showed no inhibition after 7 days against *F. graminearum* and *F. oxysporum*, respectively. The isolates MYSN 10, MYSN 109, and MYSN 18 failed to inhibit *A. flavus*. The other isolates showed varied antifungal activities as reported in Table 2.

**TABLE 2:** Antifungal activity of LAB strains isolated from Neera samples against mycotoxigenic fungi.

**Isolates**	**Tested fungal strains with ZOI (in mm)**					
	***F. graminearum***		***A. flavus***		***F. oxysporum***	
	**2 days**	**7 days**	**2 days**	**7 days**	**2 days**	**7 days**
MYSN 10	+	+	+	−	+++	+
MYSN 106	+++	+++	+++	++	+	+
MYSN 43	+	+	+	−	+	+
MYSN 109	+	−	+	−	+	−
MYSN 98	+++	+++	+	+	+++	+
MYSN 18	+	+	+	−	+	−
MYSN 28	+++	+++	+++	+	+++	+++

*ZOI, zone of inhibition; −, no effect detected; +, diameter of ZOI between 1 and 5 mm; ++, diameter of ZOI between 5 and 10 mm; +++, diameter of ZOI between 10 and 25 mm.*

#### Molecular Identification by 16S rDNA Sequencing and Phylogenetic Analysis

**FIGURE 3:**
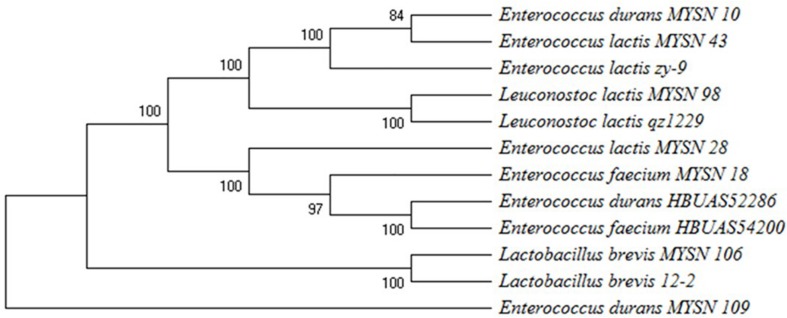
Phylogenetic tree showing the relative positions of LAB isolates (MYSN 10, MYSN 43, MYSN 98, MYSN 28, MYSN 18, MYSN 106, and MYSN 109) from Neera samples in comparison to reference strains as referred by the maximum parsimony analysis of 16S rDNA conducted in Mega X software. The percentage of replicate trees in which the associated taxa are clustered together in the bootstrap test of 500 replicates is shown next to the branches.

The potential 7 from 40 isolates, showing eminent probiotic properties with highest antimicrobial activity, were identified by 16S rDNA sequencing and phylogenetic analysis as reported in Figure 3. The isolate MYSN 106, identified as *Lactobacillus brevis* with accession no. MH748630, proved to have excellent probiotic properties. The other LAB isolates were identified as *Enterococcus durans* (two strains, i.e., MH748609-MYSN 10 and MH748633-MYSN 109), *Leuconostoc lactis* (MH748629-MYSN 98), *Enterococcus lactis* (two strains, i.e., MH748625-MYSN 43 and MH748621-MYSN 28), *and Enterococcus faecium* (MH748610-MYSN 18).

#### Antibiotic Susceptibility Test

The seven selected LAB isolates were tested for their antibiotic susceptibility against different antibiotics procured from Hi Media, India. For appropriate selection of functional strains, two groups of antibiotics are generally recommended in EFSA guidelines such as inhibitors of cell wall synthesis (ampicillin and vancomycin) and inhibitors of protein synthesis (chloramphenicol, gentamycin, clindamycin, erythromycin, streptomycin, kanamycin, and tetracycline). The results obtained were compared with the zone size interpretative chart provided in the catalog. In this study, the tested isolates were susceptible toward tetracycline and streptomycin. For most of the strains, chloramphenicol, vancomycin, and streptomycin were effective inhibitors. A variable antibiotic sensitivity was observed in all the isolates and is reported accordingly in Table 3.

**TABLE 3:** Antibiotic susceptibility of LAB strains isolated from Neera samples.

**Antibiotics**	**LAB isolates**						
	**MYSN 10**	**MYSN 106**	**MYSN 43**	**MYSN 109**	**MYSN 98**	**MYSN 18**	**MYSN 28**
Ampicillin (10 μg/disc)	R	S	R	R	S	R	R
Vancomycin (30 μg/disc)	S	n.r.	S	S	n.r.	S	S
Gentamicin (10 μg/disc)	R	S	R	S	S	R	S
Kanamycin (30 μg/disc)	MS	R	R	R	R	R	R
Streptomycin (10 μg/disc)	MS	S	S	MS	S	S	S
Erythromycin (15 μg/disc)	R	S	R	R	MS	R	R
Clindamycin (2 μg/disc)	S	R	R	S	R	R	R
Tetracycline (30 μg/disc)	S	S	S	S	S	S	S
Chloramphenicol (30 μg/disc)	R	S	S	S	S	R	S

*R, resistant; S, sensitive; MS, moderately sensitive; n.r., not recommended as per EFSA guidelines. The breakpoints for the antibiotic sensitivity/resistant in mm zone of inhibition: Amp and Van (≥17/≤14); Kan and Chl (≥18/≤12); Cli and Tet (≥19/≤14); Ste and Gen (≥15/≤12); and Ery (≥23/≤13).*

#### Resistance to Phenol

The viable count of the LAB isolates was obtained on plating the MRS broth supplemented with phenol (0.4 and 0.6%) after 24 h of incubation. The phenol concentrations had a slight inhibitory effect in comparison to the MRS control without phenol, having a viable count more than 7.75 log CFU/ml. A viable count ranging from 7.75 to 9.28 log CFU/ml was observed with 0.6% phenol and 8.07 to 9.43 log CFU/ml with 0.4% phenol, while the viable count range was 9.23–10.26 log CFU/ml without phenol. The isolate MYSN 43 was the most tolerant to phenol with 9.43 and 9.28 log CFU/ml viable counts at 0.4 and 0.6% phenol, respectively.

#### Cell Surface Properties of LAB Isolates and Their Adhesion Ability to Chicken Epithelial Cells

The isolates were tested for their cell surface hydrophobicity to estimate their adhesion ability, using the hydrocarbon xylene. The isolates showed different hydrophobicities: MYSN 106 showed the highest hydrophobicity at 77.82% followed by MYSN 98 and MYSN 28 showing hydrophobicities of 71.59 and 66.35%, respectively. Further, the remaining isolates showed a varied degree of hydrophobicity with MYSN 43 showing the least hydrophobicity of 51.10%. The isolate MYSN 106 showed the highest auto aggregation at 78.95% in comparison to the other LAB isolates tested. Further, all the other isolates showed an auto aggregation between 50.29 and 69.28%.

**FIGURE 4:**
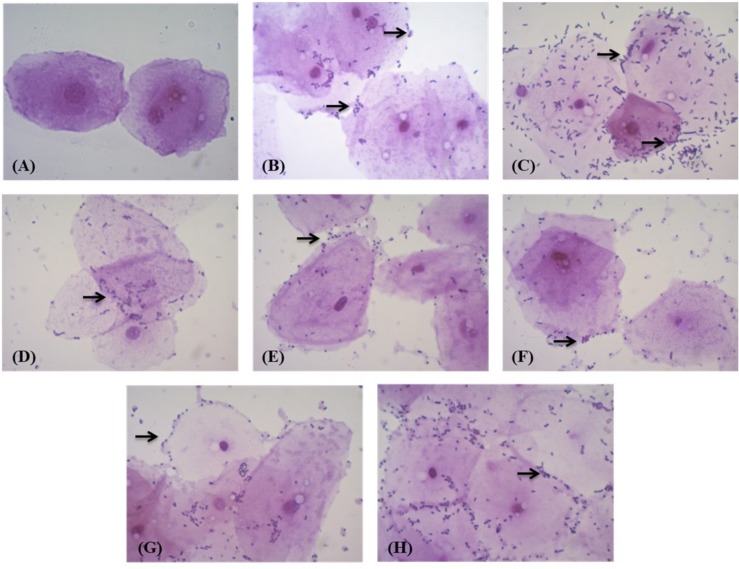
Adhesion of LAB strains to chicken crop epithelial cells observed under a light microscope. **(A)** Control epithelial cells. **(B)** Adhesion of MYSN 10 to the chicken epithelial cells. **(C)** Adhesion of MYSN 106 to the chicken epithelial cells. **(D)** Adhesion of MYSN 43 to the chicken epithelial cells. **(E)** Adhesion of MYSN 109 to the chicken epithelial cells. **(F)** Adhesion of MYSN 98 to the chicken epithelial cells. **(G)** Adhesion of MYSN 18 to the chicken epithelial cells. **(H)** Adhesion of MYSN 28 to the chicken epithelial cells.

When tested for adhesion to chicken epithelial cells, MYSN 106 showed the highest adhesion ability with 50–100 bacterial cells per epithelial cell. The MYSN 109 isolate exhibited least adhesion with 10–15 cells attaching to an epithelial cell. The varying adhesion properties observed in the other isolates are displayed in Figure 4.

#### Survival Under Gastric and Pancreatic Digestion

The isolates were tested for their colonization in the gastrointestinal tract by evaluation of their survival in simulated gastric and pancreatic digestion environments. All the isolates examined survived in both gastric and pancreatic digestion, which helps in colonizing the intestines. The viable cell count of the isolates showed that there was a minimal decrease in the viability of a few isolates after a 3-h incubation period. The MYSN 98 isolate showed the maximum viability at 3.35 × 10^6^ CFU/ml. The other isolates showed intermittent survival abilities with the viable cell count ranging from 1.45 to 2.7 × 10^6^ CFU/ml.

#### Safety Evaluation of Isolates

The safety evaluation of the isolates was determined by their hemolytic and DNAse activities, which proves the non-pathogenic status of the probiotic isolates. The results revealed no hemolytic or DNAse activities, which was confirmed by the “no zone” in the test plates inoculated with all the isolates studied.

#### Hydroxyl Radical-Scavenging Activity of Probiotic Strains

**FIGURE 5:**
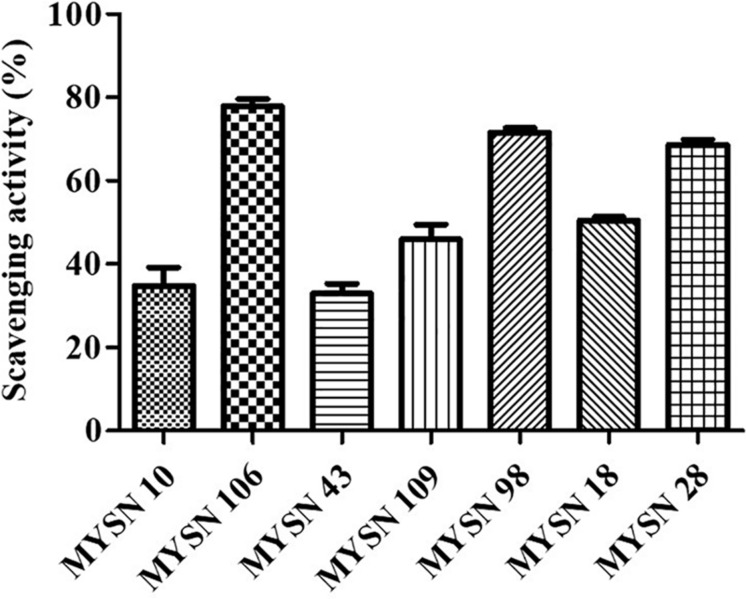
Hydroxyl radical scavenging activity of LAB strains isolated from Neera samples. Data shown are mean ± SD of triplicate values of independent experiments.

The MYSN 106 isolate showed the highest hydroxyl radical-scavenging activity at 77.87% followed by MYSN 43 and MYSN 28 with 71.51 and 68.60% hydroxyl-scavenging, respectively. The hydroxyl radical-scavenging of other isolates ranged between 28.36 and 50.47% as reported in Figure 5.

## Discussion

In this study, the importance of the selection of probiotic bacteria from traditional fermented food that can survive the human gastrointestinal tract and confer health benefits is emphasized. To isolate functional probiotic bacteria, samples of a natural fermented drink – Neera – were collected and different morphotypes were identified with their probiotic characteristics (Eckburg et al., 2005). Out of 40 bacterial strains from different Neera samples, 16 isolates survived preliminary screening for LAB strains. The presumptive LAB isolates were further characterized for acid and bile tolerances to check their viability under gastrointestinal pH conditions (Gu et al., 2008). This resulted in a display of distinct tolerance to acid and bile conditions without any significant loss in cell count with good probiotic properties. A tolerance to phenol was observed in the LAB isolates; this confers their natural selection in non-debittered Neera, which is naturally fermented with different microbial and chemical processes. This phenol tolerance is important for isolates to survive the gastrointestinal conditions, where the gut bacteria have the ability to deaminate aromatic amino acids that are derived from dietary proteins and may lead to formation of phenols (Yadav et al., 2016; Divisekera et al., 2019; Singhal et al., 2019). There are many instances of phenol tolerance reported in LAB that were isolated from natural fermented food sources (Ghabbour et al., 2011). The results prove that the isolates evaluated in the present study can survive human gastrointestinal conditions.

The isolates were further tested for antimicrobial activity. The isolates MYSN 106, MYSN 98, and MYSN 28 showed the highest antibacterial activity against the enteric pathogens tested. The obtained results clearly show the role of organic acids for the antagonistic activity of the isolates tested. The increased production of the organic acids through the fermentation reduces the pH of the media, which is known to inhibit the pathogens reducing their intercellular pH, leading to disruption in vital cell functions (Kivanç and Yilmaz, 2011). The MYSN 106 isolate proved to have the best antifungal activity for the tested mycotoxigenic fungi. The inhibitory effect, due to their competitive exclusion to bind to the gastrointestinal tract, is essential for the selection of probiotic organisms as starter cultures; this is generally associated with antimicrobial metabolites and antifungal compounds (Henning et al., 2015; Son et al., 2017). Thus, the species used as a probiotic starter culture may play an important role in providing health benefits to consumers and also in avoiding food spoilage due to colonization of mycotoxigenic fungi (Wei et al., 2006; Guimarães et al., 2018). These isolates can also be used in agricultural practices for controlling mycotoxigenic fungi in post- and pre-harvesting practices (Nagaraja et al., 2016; Deepa et al., 2018).

The LAB isolates from Neera samples are resistant to some of the antibiotics tested in the present study. The results of antibiotic susceptibility are similar to previous studies that have also reported the absence of acquired resistance in the LAB that were isolated from natural fermented samples (Casado et al., 2014). Even though certain antibiotic-resistant, infectious strains of enterococci, including *E. faecium*, have been identified, they very rarely present a risk of infection outside healthcare situations (Sanders et al., 2010). However, enterococci strains of plant origin generally display low levels of virulence, as they colonize GIT producing bacteriocins and by the fact that they have been used safely for years as probiotics in humans, and farm animals (Arias and Murray, 2012; Hanchi et al., 2018). Also, the LAB frequently harbor plasmids of different sizes, and some may contain antibiotic determinants. Therefore, if the LAB including enterococci isolates are used as starter cultures, they exhibit virulence only if the organism has the ability to transfer the resistance. Before using these isolates in food or feed formulations as per EFSA guidelines, the virulence and antimicrobial resistance genes will be verified to prevent the horizontal gene transfer for antibiotic resistance (Rychen et al., 2018). A pre-market safety assessment is also required where the safety of the isolates is assessed at species level (Montealegre et al., 2016; Divisekera et al., 2019).

The cell surface hydrophobicity and auto aggregation experiments help in studying the colonization and adhesion of probiotic bacteria to epithelial cells in the gastrointestinal tract, which lead to the prevention of colonization by pathogens through their interaction (Abushelaibi et al., 2017). The results obtained show that all the isolates have comparable auto aggregation ranging from 40 to 80% and hydrophobicity ranging from 50 to 75%, with MYSN 106 proving to be the best after 2 h of incubation at 37°C. The adhesion to chicken epithelial cell experiment also showed results comparable to those of auto aggregation and hydrophobicity; good adhesion was exhibited through interaction of cell surface components, which is one of the important probiotic characteristics (Kumari et al., 2016).

The simulated gastric and pancreatic digestion was done to test the survival of LAB isolates under the harsh conditions present in the gastrointestinal tract. The isolates could sustain the simulated digestive conditions without any loss in the viable cell count. Safety evaluation through DNase test to check for the pathogenicity of bacteria producing DNase enzyme that may cause hydrolysis of the DNA molecules was performed. Therefore, the absence of DNase in antimicrobial strains tested was confirmed to support the safety of their use in fermentations (Yadav et al., 2016; Singhal et al., 2019). The hydroxyl radical-scavenging activity of the LAB isolates is due to the colonization of viable cells and their propagation in the gut. The results obtained are comparable with previous studies as they also confer that the isolates tested help in the cure of cardiovascular diseases and gastrointestinal disorders and also improve the immune response (Kaushik et al., 2009). Among all the isolates tested, the isolate MYSN 106, which exhibited the highest potential as a probiotic, was identified as *L. brevis* by 16S rDNA sequencing and phylogenetic analysis with 99% homology. Many studies have been conducted demonstrating the probiotic potential of *L. brevis* isolated from a wide variety of fermented food samples (Pennacchia et al., 2004).

The LAB strains (MYSN 10, MYSN 106, MYSN 43, MYSN 109, MYSN 98, MYSN 18, and MYSN 28) isolated from the naturally fermenting Neera demonstrated probiotic attributes *in vitro* with good antimicrobial properties, proving their potential to be used as a starter culture in fermented food products, food or feed preservation, scavenging pathogens, and the biocontrol of mycotoxigenic fungi.

## Conclusion

The probiotic strains that are isolated from traditional fermented food have a broad spectrum of antimicrobial activity and can be used as preservatives in food products. In this work, the authors focused on screening probiotic bacteria from the naturally fermented beverage – Neera. All the seven isolates exhibited resistance to gastrointestinal conditions and good antimicrobial activity. Among the seven isolates selected on screening, *L. brevis* MYSN 106 proved to be best – surviving the low pH and bile conditions in the stomach, including the harsh intestinal conditions. It also possesses surface-binding properties capable of colonizing the gastrointestinal tract, which is important for antimicrobial activity and disease treatment; this makes it a potential probiotic. Finally, the LAB isolates from Neera demonstrated probiotic attributes with good antimicrobial activities *in vitro*, therefore exhibiting potentiality to use them as probiotics in food and feed formulations.

## Author Contributions

MYS and RS designed the research. RS, BS, and BVD carried out the research activities. RS analyzed the data and wrote the manuscript. BVD and MYS edited and submitted the final version of the manuscript. All authors gave their approval for publication.

## Conflict of Interest Statement

The authors declare that the research was conducted in the absence of any commercial or financial relationships that could be construed as a potential conflict of interest.
